# Bilateral vessel-outlining carotid artery calcifications in panoramic radiographs: an independent risk marker for vascular events

**DOI:** 10.1186/s12872-019-1211-3

**Published:** 2019-10-16

**Authors:** Maria Garoff, Jan Ahlqvist, Linda-Tereza Edin, Sofia Jensen, Eva Levring Jäghagen, Fredrik Petäjäniemi, Per Wester, Elias Johansson

**Affiliations:** 1 Department of Odontology/Oral and Maxillofacial Radiology, Umeå University, SE-901 87 Umeå, Sweden; 2 Department of Public Health and Clinical Medicine, Umeå University, Umeå, Sweden; 3 Department of Pharmacology and Clinical Neuroscience, Umeå University, Umeå, Sweden; 4 Department of Clinical Sciences, Danderyd hospital, Stockholm, Sweden; 5 Wallenberg Centre for Molecular Medicine, Umeå University, Umeå, Sweden

**Keywords:** Panoramic radiography, Carotid atherosclerosis, Cardiovascular disease

## Abstract

**Background:**

In odontology, panoramic radiographs (PRs) are regularly performed. PRs depict the teeth and jaws as well as carotid artery calcifications (CACs). Patients with CACs on PRs have an increased risk of vascular events compared to healthy controls without CACs, but this association is often caused by more vascular events and risk factors at baseline. However, the risk of vascular events has only been analyzed based on the presence of CACs, and not their shape. Thus, this study determined if the shape of CACs in PRs affects the risk of future vascular events.

**Methods:**

The study cohort included 117 consecutive patients with CACs in PRs and 121 age-matched controls without CACs. CAC shape in PRs was dichotomized into bilateral vessel-outlining CACs and other CAC shapes. Participants were followed prospectively for an endpoint of vascular events including myocardial infarction, stroke, and vascular death.

**Results:**

Patients with bilateral vessel-outlining CACs had more previous vascular events than those with other CAC shapes and the healthy controls (*p* < 0.001, χ^2^). The mean follow-up duration was 9.5 years. The endpoint was reached in 83 people. Patients with bilateral vessel-outlining CACs had a higher annual risk of vascular events (7.0%) than those with other CAC shapes (4.4%) and the controls (2.6%) (*p* < 0.001). In multivariate analysis, bilateral vessel-outlining CACs (hazard ratio: 2.2, 95% confidence interval: 1.1–4.5) were independent risk markers for the endpoint.

**Conclusions:**

Findings of bilateral vessel-outlining CACs in PRs are independent risk markers for future vascular events.

## Background

Atherosclerosis is the main cause of vascular events [1]. Because it is a slow, progressive disease, there is opportunity to introduce preventive treatments in targeted subgroups with high vascular risk [2]. One strategy for distinguishing subgroups with high vascular risk is the identification of atherosclerotic carotid plaques. These plaques, even when causing less than 50% stenosis, are associated with an increased risk of vascular events such as myocardial infarction (MI), stroke, and vascular death [3]. Population-level risk assessments of subclinical asymptomatic atherosclerotic carotid plaques that require minimal invasiveness are difficult to achieve because intra-plaque C-reactive protein, carotid plaque morphology, and carotid plaque burden are markers that require expensive and sometimes invasive screening methods; thus, supplemental methods are warranted [2, 4–11]. One supplemental method may be to screen for carotid artery calcifications (CACs) in panoramic radiographs (PRs) performed in patients for odontological reasons.

PRs are frequently performed in general and specialized dental care in all age groups. They are two-dimensional images specifically developed for examination of the teeth and jaws, but they also depict parts of the cervical soft tissues including the carotid arteries. CACs are found in 3–15% of PRs, and 99% of CACs coincide with ultrasound-verified calcification within an atherosclerotic plaque ≥1 mm^3^ [12–15]. The prevalence of CACs increases with age and among individuals with previous vascular events and/or vascular risk factors [12, 13]. In all studies including > 20 individuals with CACs, those with CACs had an increased risk of future vascular events compared to healthy controls without CAC [16, 17] or controls not examined with PRs [18]. However, the association between CACs and future vascular events was not independent when adjusting for baseline characteristics [16, 17].

The association between CAC shape and vascular risk has never been assessed. CACs are divided into single, spread, and vessel-outlining shapes. A single CAC comprises one single calcification; scattered CACs comprise several smaller and spread calcifications; and vessel-outlining CACs comprise single or scattered CACs that give the impression of outlining the contours of the carotid artery [19]. Vessel-outlining CACs are associated with ≥50% carotid stenosis and can be identified with high inter-rater reliability (kappa 0.78) [19]. We hypothesized that vessel-outlining CACs in PRs are independent risk markers for future vascular events.

The aim of this study was to determine if the shape of CACs in PRs affects the risk of future vascular events.

## Methods

### Study population

**Fig. 1:**
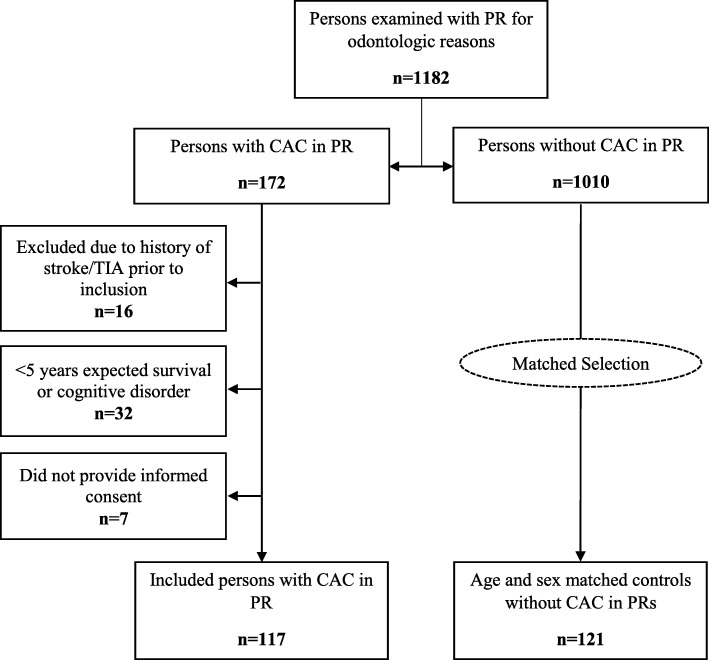
Flow chart showing the distribution of persons with and without CACs and exclusions. PR: Panoramic radiograph. CAC: Carotid artery calcification (detected on panoramic radiographs). TIA: Transient ischemic attack

The study population has been previously described [12, 16, 19]. In brief, between August 2007 and February 2009, we examined 1182 patients who had PRs performed for odontological reasons at the Department of Oral and Maxillofacial Radiology, Umeå University (Umeå, Sweden). Only patients between 18 and 74 years of age were included, as this study was originally designed to detect individuals with asymptomatic carotid stenosis eligible for carotid endarterectomy, and one criterion for treatment was age < 75 years. We included consecutive patients with CACs in PRs and a similar number of healthy controls without CAC, matched for age and sex. The study initially comprised 450 patients with CAC or matched controls without CAC. However, 212 persons were excluded including 157 controls. Exclusion criteria were: history of stroke or transient ischemic attack (TIA), serious concomitant diseases resulting in < 5-year expected survival (due e.g. cancer), cognitive disorders, and/or not providing informed consent. A total of 238 participants were included in the study including 117 cases with CAC and 121 controls without CAC (Fig. 1). All study participants provided written informed consent, and the study was approved by the Regional Ethical Review Board in Umeå, Sweden (No. 07–044 M).

### Radiographic examination and evaluation

**Fig. 2:**
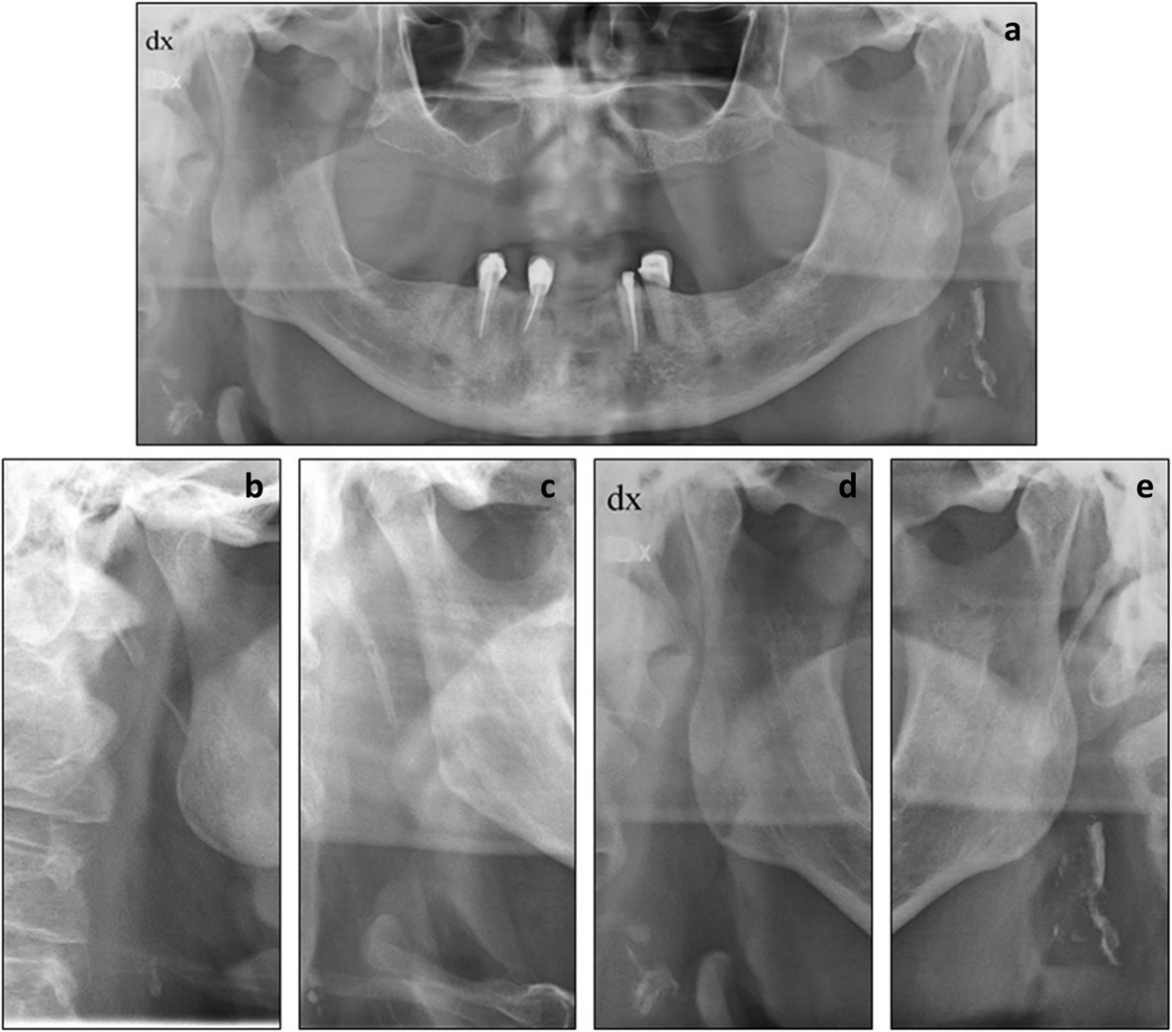
**a**. Panoramic radiograph illustrating bilateral vessel-outlining carotid artery calcifications (CACs), enlarged in **d** and **e**. **b** and **c** cropped panoramic radiographs illustrating single (**b**) and scattered (C) CACs

Digital panoramic examinations of the jaws were performed according to standard procedures as described in Johansson et al. [12] Two specialists in Oral and Maxillofacial Radiology (JA, ELJ), who were blinded to the medical records, re-evaluated all PRs with CACs regarding shapes defined as single, spread, or vessel-outlining (Fig. 2) [19]. In cases of disagreement regarding shape, the two examiners reached consensus after discussion.

In an exploratory step, study participants were initially divided into six groups according to CAC shape in the PR exam: (1) none (controls without CAC), (2) unilateral single/spread, (3) bilateral single/spread, (4) unilateral vessel-outlining, (5) bilateral CAC including one vessel-outlining, and (6) bilateral vessel-outlining. Based on the outcome of this explorative step, groups were merged in the main analysis.

### Data collection

Baseline characteristics were assessed at the time of PR examination. The presence of risk factors was determined from documentation in the medical records. If there was no documentation about risk factors in the medical records, the data were regarded as missing. In an explorative analysis, medical prevention was categorized as “easily improved by medical intervention” if a person lacked ≥1 type of medical prevention (i.e. lacked an anti-platelet and anti-coagulant, blood pressure reducing medication, and/or combination therapy of a statin with ezetimibe). Prospective follow-up was performed by reviewing surveys, telephone, and medical records. The last follow-up was conducted for all participants in 2018.

### Endpoints

The primary endpoint was a combination of vascular events occurring after inclusion including stroke, TIA, MI (ST-segment and non-ST-segment elevation MI), new-onset heart failure with clear clinical diagnosis, new-onset angina pectoris with clear clinical diagnosis, new-onset symptomatic claudication of Leriche IIA or worse, arterial revascularization, and vascular death (vascular heart diseases, stroke, and severe diabetic vascular complications). Secondary endpoints were all separate components of the primary endpoint and all-cause mortality. For patients with several types of vascular events, the first was used as the primary endpoint, but all were also assessed separately as secondary endpoints.

### Statistical analyses

We used mean, median, standard deviation (SD), interquartile range, 95% confidence interval (CI), two-sided χ^2^-test, and Kruskal-Wallis test as specified. Median was used for age, as it was not normally distributed because we had an upper age limit (< 75 years). *P* values < 0.05 were considered statistically significant. The endpoints were analyzed with the Kaplan-Meier method (log-rank test) and Cox regression model, using end of follow-up, lost to follow-up, and non-vascular death as censors. In the cox-regression, all co-variates were evaluated for collinearity (all factors were checked against all others), curve crossing for and interaction with the primary endpoint, without any relevant finding. All baseline characteristics except medical treatment were co-variates in multivariate analysis, as medical treatment reflects management whereas remaining factors reflect the state of health. All co-variates were analyzed for association with the primary endpoint (bivariate analysis), and then entered into a multivariate model. The co-variate with the highest *p*-value was stepwise removed until all had *p* < 0.05. Annual risk was assessed by reading the Kaplan-Meier curve at 9.5 years (mean follow-up). IBM SPSS statistics 25 statistical software was used for all statistical analyses. EJ had full access to all data in this study and was primarily responsible for data integrity and analysis.

## Results

The distribution of CAC shapes was 12% (14/117) unilateral single/spread, 26% (31/117) bilateral single/spread, 10% (12/117) unilateral vessel-outlining, 30% (35/117) bilateral CAC including one vessel-outlining, and 21% (25/117) bilateral vessel-outlining.

**Fig. 3:**
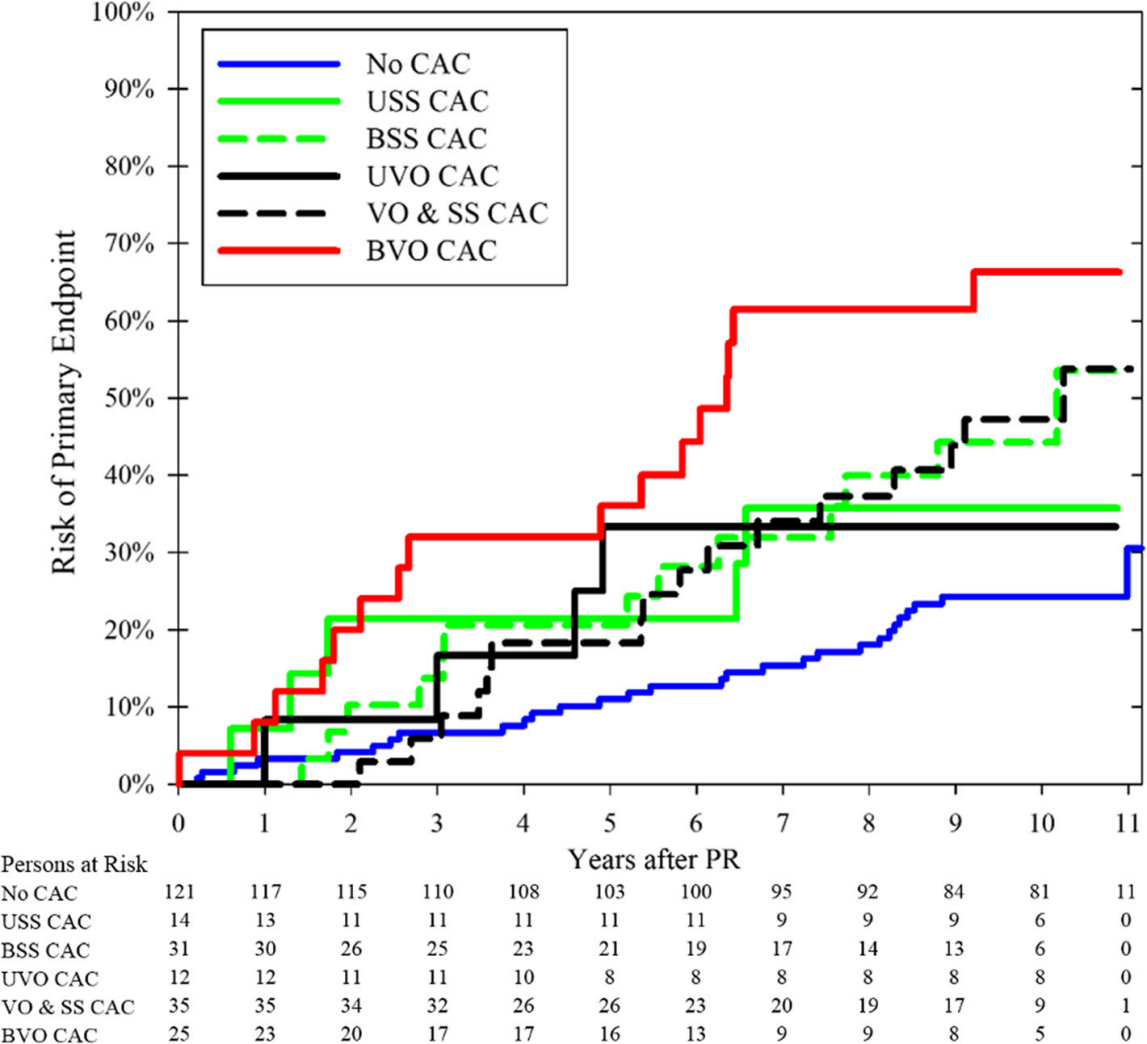
Kaplan-Meier analysis comparing the risk of the primary endpoint between all six CAC groups. Significant differences noted between the groups (*p* < 0.001, log rank test). Censoring at lost to follow-up and non-vascular death. CAC: Carotid artery calcification (detected on panoramic radiographs). USS: Unilateral single/spread. BSS: Bilateral single/spread. UVO: Unilateral vessel-outlining. VO & SS: Bilateral CAC, of which one vessel-outlined and one single/spread. BVO: Bilateral vessel-outlining

**Fig. 4:**
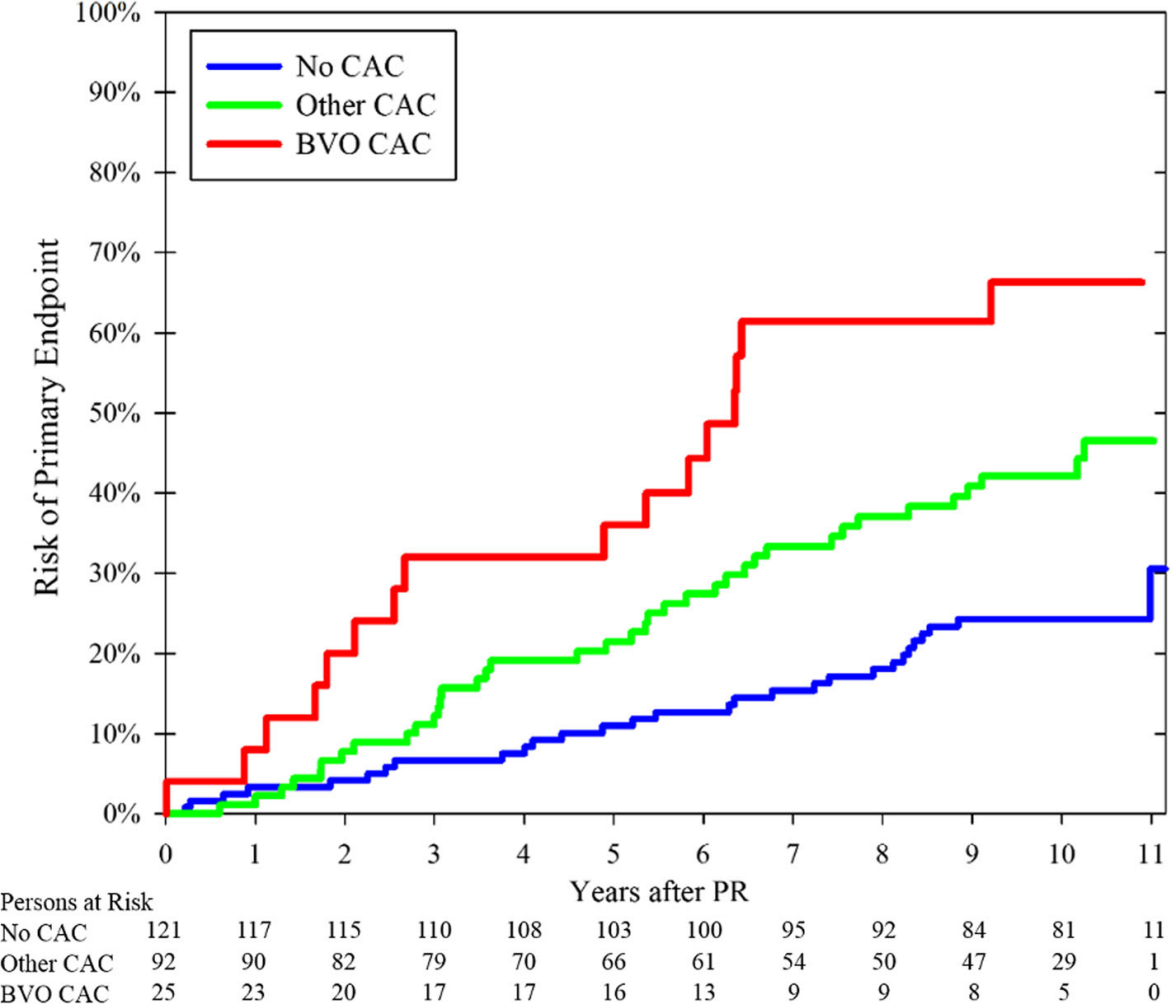
Kaplan-Meier analysis comparing the risk of the primary endpoint between the three main CAC groups. Significant differences noted between the groups (p < 0.001, log rank test). Censoring at lost to follow-up and non-vascular death. CAC: Carotid artery calcification (detected on panoramic radiographs). BVO: Bilateral vessel-outlining

The mean follow-up duration was 9.5 years (SD: 2.4, range: 0.6–11.1). A total of 3 (3%) cases and 3 (3%) controls were lost to follow-up after a mean of 5.4 years (SD: 2.6), 50 cases were followed until death for a mean of 6.1 years (SD: 2.9), and the remaining 182 cases were followed until last follow-up for a mean of 10.5 years (SD: 0.4, range: 9.4–11.1). During follow-up, 83 patients reached the primary endpoint with an average annual risk of 3.7% (95% CI: 3.1–4.4%). The risk of meeting the primary endpoint was 2.6%/year for patients without CACs. All CAC groups except the bilateral vessel-outlining group had a similar intermediate annual risk of meeting the primary endpoint (3.5–5.0%/year); the bilateral vessel-outlining had a high annual risk of 7.0%/year (Fig. 3). Consequently, all CAC groups except the bilateral vessel-outlining group were combined into a single “other CACs” group in subsequent analyses (Fig. 4).

Patients with bilateral vessel-outlining CACs were slightly older and had more previous vascular events (current angina, previous MI, previous arterial revascularization) than those with other CACs and controls without CAC (Table 1).

**Table 1:** Baseline comparisons. All differences between groups were analyzed with 2-sided χ^2^ test except for age, which was analyzed with Kruskal-Wallis test

	Missing data	Controls without CAC (*n* = 121)	Other CACs (*n* = 92)	Bilateral vessel-outlining CACs (*n* = 25)	*P* value
Age, median (IQR)	0	67 (63–70)	66 (63–71)	70 (68–73)	0.011
Men, n (%)	0	64 (53)	51 (55)	12 (48)	0.80
Myocardial infarction, n (%)	1	7 (6)	15 (16)	8 (32)	0.001
Current heart failure, n (%)	1	2 (2)	8 (9)	2 (8)	0.052
Current angina pectoris, n (%)	1	14 (12)	23 (25)	9 (36)	0.004
Current symptomatic claudication, n (%)	1	2 (2)	3 (3)	1 (4)	0.87
Revascularization, n (%)	1	8 (7)	18 (20)	6 (24)	0.006
Any vascular event, n (%)	1	20 (17)	36 (40)	13 (52)	< 0.001
Current smoking, n (%)	1	11 (9)	23 (25)	5 (20)	0.007
Diabetes, n (%)	1	12 (10)	27 (30)	5 (20)	0.001
Atrial fibrillation, n (%)	11	6 (5)	5 (6)	1 (4)	1.0
Valvular heart disease, n (%)	12	7 (6)	10 (11)	5 (21)	0.07
Hypertension*, n (%)	16	79 (71)	73 (85)	22 (92)	0.013
Hyperlipidemia†, n (%)	46	80 (88)	68 (87)	23 (100)	0.21
Any risk factor, n (%)	1	105 (87)	87 (96)	24 (96)	0.049
Easily improved by medical prevention‡, n (%)	0	102 (84)	68 (74)	14 (56)	0.006

CAC: Carotid artery calcification (detected on panoramic radiographs)

IQR: Interquartile range

*Recorded blood pressure of > 140/90 mmHg and/or prescription of common blood pressure reducing medication (alpha-blocker not included)

†Recorded cholesterol ≥5.0 mmol/l and/or prescription of statin and/or Ezetimibe

‡ Lacking ≥1 type of medical prevention: (1) an anti-platelet or anti-coagulant, (2) blood pressure reducing medication and (3) Statin and/or Ezetimibe

The risk of meeting the primary endpoint (combined vascular events endpoint) significantly differed among the three CAC groups (*p* < 0.001; Fig. 4). The risk was higher for bilateral vessel-outlining CACs compared to other CACs (*p* = 0.024) and was also higher for other CACs compared to controls without CAC (*p* = 0.002). New-onset heart failure, arterial revascularization, and vascular death accounted for the increased risk of bilateral vessel-outlining CACs (Table 2). In addition to shape, several other baseline characteristics were associated with the primary endpoint in the bivariate analyses (Table 3). In multivariate analysis, bilateral vessel-outlining CACs (adjusted hazard ratio [HR]: 2.2, 95% CI: 1.1–4.5, *p* = 0.02) were independent risk markers for the primary endpoint.

**Table 2:** Annual risk for the primary and secondary endpoints

	Controls without CAC (n = 121) Annual risk (n; 95% CI)	Persons with other CAC (n = 92) Annual risk (n; 95% CI)	Persons with bilateral vessel-outlining CACs (n = 25) Annual risk (n; 95% CI)	Log rank test
Primary endpoint^*^	2.6% (29; 1.7–3.4%)	4.4% (38; 3.3–5.6%)	7.0% (16; 5.0–9.0%)	< 0.001
Myocardial infarction	0.6% (7; 0.2–1.1%)	1.6% (12; 0.8–2.4%)	0.7% (1; 0.0–2.0%)	0.08
New-onset heart failure	0.7% (8; 0.2–1.1%)	0.7% (7; 0.1–1.2%)	3.2% (7; 1.2–5.2%)	< 0.001
New-onset angina pectoris	0.6% (6; 0.1–1.0%)	0.5% (5; 0.0–1.1%)	0.7% (1; 0.0–2.0%)	0.93
New-onset symptomatic claudication	0.1% (1; 0.0–0.3%)	1.3% (10; 0.5–2.0%)	0.9% (2; 0.0–2.0%)	0.003
Stroke	0.9% (10; 0.4–1.4%)	0.8% (7; 0.2–1.5%)	2.4% (5; 0.5–4.3%)	0.08
Transient ischemic attack	0.4% (5; 0.0–0.7%)	0.9% (7; 0.3–1.6%)	0.4% (1; 0.0–1.3%)	0.38
Arterial revascularization	0.8% (9; 0.3–1.4%)	1.7% (13; 0.9–2.6%)	2.9%(6; 0.9–4.9%)	0.011
Vascular death†	0.2% (1; 0.0–0.7%)	1.0% (9; 0.4–1.7%)	2.2% (5; 0.5–3.9%)	< 0.001
All-cause mortality‡	1.1% (18; 0.5–1.7%)	2.6% (26; 1.7–3.6%)	4.2% (11; 2.2–2.6%)	< 0.001

CAC: Carotid artery calcification (detected on panoramic radiographs)

^*^Primary endpoint was the combination of all vascular events in the table, but not all-cause mortality

†Heart failure (*n* = 5), cardiac arrest (n = 5), myocardial infarction (n = 3), stroke (n = 1) and diabetic ketoacidosis (*n* = 1). The two none-cardiac deaths occurred in the persons with other CACs group

‡Vascular death (*n* = 15), cancer (*n* = 19), respiratory disease (*n* = 4), infection (n = 4), trauma and intoxication (*n* = 3), post-operative organ failure (n = 2), non-diabetic metabolic acidosis (n = 1), kidney failure (n = 1) and aortic dissection (n = 1)

**Table 3:** Bivariate and multivariate Cox-regression analysis of the primary endpoint

	Bivariate model before step-wise removal		Multivariate model after step-wise removal	
	Hazard ratio (95% CI)	*p*-value	Hazard ratio (95% CI)	*p*-value
Controls without CAC	1.0 (ref)	–	1.0 (ref)	–
Other CACs	1.8 (1.2–2.8)	0.003	1.4 (0.8–2.4)	0.19
Bilateral vessel-outlining CACs	3.6 (2.2–6.0)	< 0.001	2.2 (1.1–4.5)	0.02
Age (10-year increase)	1.5 (1.0–2.2)	0.048	–	–
Male sex	1.2 (0.8–1.8)	0.46	–	–
Previous myocardial infarction	3.0 (1.8–5.0)	< 0.001	2.6 (1.4–4.7)	0.002
Current heart failure	3.0 (1.5–6.3)	0.003	–	–
Current angina pectoris	1.7 (1.0–2.7)	0.044	–	–
Current symptomatic claudication	3.1 (1.1–8.5)	0.03	3.2 (1.1–9.1)	0.03
Revascularization	2.6 (1.5–4.3)	< 0.001	–	–
Any vascular event*	3.4 (2.2–5.2)	< 0.001	–	–
Current smoking	2.0 (1.2–3.3)	0.005	1.9 (1.1–3.2)	0.01
Diabetes	1.9 (1.2–3.1)	0.009	–	–
Atrial fibrillation	1.5 (0.7–3.5)	0.31	–	–
Valvular heart disease	2.6 (1.5–4.7)	< 0.001	2.3 (1.3–4.0)	0.006
Hypertension	2.1 (1.1–4.0)	0.03	–	–
Hyperlipidemia	1.9 (0.8–4.7)	0.16	–	–
Any risk factor^*^	4.7 (1.2–19.2)	0.03	–	–

^*^Not used in the multivariate model

CAC: Carotid artery calcification (detected on panoramic radiographs)

To reduce the risk of drawing incorrect conclusions, we explored two similar multivariate models: (1) excluding hypertension and hyperlipidemia as they had many missing values, and (2) only including co-variates associated (*p* < 0.1) with the primary endpoint (bivariate analysis, Table 3) or with CAC shape (Table 1); in both instances, the final model was identical to the first model.

Among patients using all three classes of assessed medications (anti-platelet, anti-coagulant, blood pressure reducing medication, and/or combination therapy of a statin with ezetimibe), there was no significant difference in annual risk of meeting the primary endpoint; specifically, the risk was 7.2% for (bilateral vessel-outlining CACs), 7.0% for (other CACs,) and 3.7% for controls lacking CACs) (*p* = 0.17). However, among patients not using all three classes of assessed medications, the annual risk of meeting the primary endpoint was significantly different, namely 6.8% for bilateral vessel-outlining CACs, 3.5% for other CACs, and 2.0% for controls lacking CACs (*p* < 0.001). We repeated the multivariate analysis in patients not using all three classes of assessed medications: For bilateral vessel-outlining CACs compared to controls, the unadjusted HR was 4.6 (95% CI: 2.1–10.0, *p* < 0.001) and the adjusted HR was 3.1 (95% CI: 1.3–7.3, *p* = 0.009). For other CACs compared to controls, the unadjusted HR was 1.9 (95% CI: 1.1–3.5, *p* = 0.03) and the adjusted HR was 1.3 (95% CI: 0.7–2.5, *p* = 0.37).

## Discussion

The main finding in this study was that bilateral vessel-outlining CACs are independent risk markers for future vascular events. This is the first time that the detailed shape of calcifications in PRs has been linked to risk for future vascular events. PRs are performed for odontological reasons. CACs can be identified and differentiated into shapes in an examination that is not expensive, time consuming, or invasive. These results have clinical utility, as they provide a novel cardiovascular risk marker of minimum cost that can be used in clinical practice.

We found that the presence of all shapes of CACs in PRs was associated with future vascular events, but only bilateral vessel-outlining CACs were independently associated with these events. The mechanisms underlying these findings are unclear, as no studies have analyzed the potential differences among single, spread, and vessel-outlining CACs; however, it seems reasonable that the mechanism is the identification of variations of advanced atherosclerosis. In ultrasound studies, a large carotid plaque burden (plaque area/volume or scores including < 50% stenosis) is associated with vascular events, which are usually non-stroke events [6–10]. Hence, rather than being causative emboli sources, they are associated with risk by marking advanced atherosclerosis. Similarly, we found that for bilateral vessel-outlining CACs, non-stroke events such as cardiac vascular death caused an increased risk of vascular events. We also found that bilateral vessel-outlining CACs seemed to result in a higher risk of vascular events than unilateral vessel-outlining CACs, indicative of more advanced atherosclerosis. In addition, patients with bilateral vessel-outlining CACs had an increased number of previous vascular events at baseline, which is expected in people with more advanced atherosclerosis. The fact that the vast majority of patients with bilateral vessel-outlining CACs have < 50% carotid stenosis [19] is not a compelling counterargument as ultrasound plaque burden studies also include < 50% stenosis [6–10]. Thus, comparisons between bilateral vessel-outlining CACs and plaque burden with ultrasound are warranted. As much more prognostic data are available for plaque burden than bilateral vessel-outlining CACs, it seems reasonable that screening for CACs in PRs performed for odontological reasons might be a pragmatic approach for identifying people with bilateral vessel-outlining CACs. These patients could be further examined by carotid ultrasound imaging for the identification of high plaque burden.

Our finding that bilateral vessel-outlining CACs are independent risk markers for future vascular events suggest that medical intervention might be indicated. The fact that bilateral vessel-outlining CACs, in contrast to other CACs, are independent of baseline characteristics is relevant as it contributes additional information to the field and does not merely reflect what is already known. This finding remained in the exploratory analysis, when limiting the analysis to those not using all three classes of assessed medications at baseline. This finding is encouraging as this group can easily receive intensified medical treatment. As PR is well-used in developed countries, the annual number of incidental findings of bilateral vessel-outlining CACs is likely to be substantial, and each might be an opportunity to prevent future vascular events. Therefore, it is worthwhile to further investigate if intensive medical treatment causes a reduction in vascular events in patients with bilateral vessel-outlining CACs.

The strengths of this study were the long-term follow-up, prospective design, consecutively sampled population, age- and sex-matched controls, and high inclusion rate. Study limitations were that due to the primary inclusion criteria, it only included participants < 75 years eligible for asymptomatic carotid endarterectomy and those lacking a history of stroke/TIA. Vascular risk factors were measured by several healthcare providers, according to standard procedures. The definition “easily improved by medical intervention” did not include glucose-lowering therapy, as all patients had not undergone assessment for possible diabetes. The original study design did not include coronary artery calcification scores. CAC shape in PRs as described in this study were not further analyzed with other methods, and the difference between vessel-outlining CAC and other shapes in relation to ultrasound-assessed shapes of carotid plaques remains unclear. Further, this study was observational and hypothesis-generating; thus, confirmatory studies and randomized controlled trials of medical prevention are warranted to confirm these findings.

## Conclusions

Bilateral vessel-outlining CACs in PRs are independent risk markers for future vascular events. Future studies should assess if intensive medical intervention is needed in patients with bilateral vessel-outlining CACs.

## Data Availability

The datasets used and/or analysed during the current study are available from the corresponding author on reasonable request.

## Abbreviations

CACs: Carotid artery calcifications
PR: Panoramic radiograph
TIA: Transient ischemic attack
